# Nano-enabled crop resilience against pathogens: potential, mechanisms and strategies

**DOI:** 10.1007/s44297-023-00015-8

**Published:** 2023-11-30

**Authors:** Muhammad Noman, Temoor Ahmed, Jiaoyu Wang, Munazza Ijaz, Muhammad Shahid, Mohammad Shafiqul Islam, Azizullah, Irfan Manzoor, Dayong Li, Fengming Song

**Affiliations:** 1 Ministry of Agriculture Key Laboratory of Molecular Biology of Crop Pathogens and Insects and State Key Laboratory of Rice Biology and Breeding, Institute of Biotechnology, College of Agriculture and Biotechnology, Zhejiang University, Hangzhou, 310058 China; 2 State Key Laboratory for Managing Biotic and Chemical Treats to the Quality and Safety of Agro-Products, Institute of Plant Protection and Microbiology, Zhejiang Academy of Agricultural Sciences, Hangzhou, 310021 China; 3 Xianghu Laboratory, Hangzhou, 311231 China; 4 Department of Bioinformatics and Biotechnology, Government College University, Faisalabad, 38000 Pakistan

**Keywords:** Antimicrobial activity, Crop disease management, Nanoparticles, Plant immunity, Sustainable agriculture

## Abstract

Nanoparticles (NPs) have emerged as a revolutionary strategy in the field of agriculture, offering innovative solutions for enhancing plant health, disease management, and sustainable crop production. This review summarizes the multifaceted roles of NPs, synthesized chemically and biologically, in crop disease management, encompassing the NP modulation of plant immunity against pathogens, mechanisms of NP uptake, and potential applications in disease control. The integration of NPs as delivery vehicles for bioactive molecules, enabling targeted delivery of nutrients, hormones, RNA interference molecules, and chemical protectants for growth regulation and disease management, is also discussed in detail. The review also critically examines the safety and environmental considerations associated with the potential application of NPs in the agriculture sector, including environmental toxicity, fate, and risks. Future perspectives encompass precision agriculture, eco-friendly disease management, unraveling intricate plant-NP interactions, and the necessity for responsible innovation. At the nexus of nanotechnology and agriculture, this review underscores the transformative potential of NPs in revolutionizing plant health and crop disease management, while highlighting the importance of responsible application to ensure sustainable and resilient agricultural systems.

## Introduction

Plant diseases caused by phytopathogens, including fungi, bacteria, viruses, and nematodes, have long been a major challenge in agriculture, posing significant threats to crop productivity and global food security [1]. The interaction between plants and phytopathogens is a complicated dynamic process, involving intricate physiological, biochemical and molecular responses [2, 3]. Traditional methods of managing plant diseases often rely on chemical pesticides, which not only pose potential risks to human health and the environment but also contribute to the development of pesticide-resistant pathogens [4, 5]. The demand for sustainable and eco-friendly agricultural practices has led researchers to explore innovative strategies that can boost the efficiency of the green control of crop diseases while minimizing the negative impacts of conventional disease management approaches.

In recent years, the field of nanotechnology has emerged as a promising avenue for revolutionizing plant disease management by harnessing the unique properties of nanoparticles (NPs) to modulate plant immunity and counteract phytopathogen attacks [6–8]. In the realm of NP-mediated disease management in crops, NPs have risen to prominence due to their unique physicochemical properties and remarkable versatility [9, 10]. In recent years, silver NPs, copper NPs, and others have garnered significant attention for their remarkable antimicrobial properties and their ability to enhance plant defenses [11, 12]. Recent research has highlighted the potential of NPs to influence and strengthen plant innate defense mechanisms, thereby triggering plant immunity and reducing disease susceptibility. For example, manganese and copper NPs, at 100 μg/mL concentration, have been shown to activate innate immune responses in watermelon plants against Fusarium wilt and bacterial fruit blotch, respectively [4, 13]. Similarly, chitosan-coated iron NPs (250 μg/mL) have been reported to boost the capacity of the antioxidative system and induce the expression of defense genes to suppress bacterial leaf blight in rice [14]. The capacity of NPs to penetrate plant tissues, interact with cell membranes, and traverse cell walls enables them to access and engage with plant systems, providing an exciting opportunity to develop innovative strategies for disease management that are both effective and environmentally sustainable [15, 16]. However, several climatic factors, soil type, pH levels, and the compatibility of NPs with other substances can significantly impact the overall success of NPs-based disease management approaches [6, 10]. Furthermore, synthesizing NPs is a crucial aspect of harnessing their potential applications in the agriculture sector [17, 18]. Among the most common methods are chemical and physical methods, which are widely used for their scalability and precision in controlling NP size and shape. However, these methods require the use of toxic chemicals, high cost, extended time periods, and high temperatures [19, 20]. Biological methods, synthesizing NPs either intracellularly or extracellularly by employing microbes or plant extracts as reducing agents, offer green synthesis options [10, 21]. The choice of synthesis method depends on the specific application and desired NP properties, highlighting the need for a tailored approach in harnessing the full potential of NPs in diverse fields.

This review highlights the multifaceted roles, mechanisms and strategies of NPs for sustainable crop disease management. This study aims to provide an in-depth understanding of the effects of NPs on different crops and the mechanisms underlying the NP-mediated modulation of plant immunity. Additionally, the review also addresses critical considerations such as NP formulation strategies for optimal delivery, safety concerns, and environmental implications. By critically evaluating the state of research in this dynamic field, we aim to shed light on the future applications of NPs or their formulations in sustainable agriculture systems.

## NPs in crop disease management

NPs have emerged as a cutting-edge and transformative tool in the realm of crop disease management. In an era where sustainable agriculture is imperative, NPs offer innovative solutions to address the complex challenges posed by diverse phytopathogens [22]. Their small size, high surface area-to-volume ratio, and tunable surface chemistry enable precise and targeted interventions [10]. One of the fundamental roles of NPs in crop disease management is their capacity to stimulate plant defense responses [23]. When NPs interact with plants, they can trigger various defense pathways, including the induction of defense genes, oxidative signaling, and phytohormone-dependent molecular events. These responses prepare plants to recognize and combat invading pathogens more effectively, providing an innate shield against diseases [24, 25]. For example, phytogenic silica NPs (100 μg/mL) have been demonstrated to activate the antioxidant system and innate defense responses in wheat plants against *Rhizoctonia solani* [26]. Similarly, chitosan-coated iron NPs, synthesized by *Bacillus aryabhattai* RNT7, have been reported to trigger the expression of PR protein- and antioxidant enzyme-encoding genes to counter bacterial leaf blight disease in rice [14]. Moreover, NPs serve as carriers for essential nutrients, facilitating their uptake and efficient utilization by plants. This not only bolsters plant health but also strengthens their ability to fend off pathogens. For example, at a 30 mg/L concentration, chemogenic sulfur NPs suppressed Fusarium wilt in tomato plants by improving *in planta* sulfur accumulation and plant biomass [27]. Similarly, chemogenic silica NPs (1500 mg/L) have been employed to enhance resistance in watermelon plants against the Fusarium wilt pathogen *Fusarium oxysporum* f. sp. *niveum*, effectively reducing disease severity by improving silicon concentration in plant tissues [15]. Furthermore, NPs can also exert direct antimicrobial effects by disrupting pathogen structures, functions, and infection ability, making them effective weapons against devastating phytopathogens. For example, 16 μg/mL of phytogenic zinc and chitosan NPs, stabilized using tomato extract, displayed significant antibacterial activity against *Xanthomonas oryzae* pv. o*ryzae*, inhibiting pathogen growth, biofilm production, and swarming motility [28]. Microscopic observations revealed that these NPs induced morphological and oxidative damage to bacterial cells, ultimately leading to pathogen death [28]. By offering a multifaceted approach to disease management, NPs contribute to sustainable farming practices by reducing the need for chemical pesticides, promoting environment-friendly agriculture, and enhancing crop productivity [29, 30].

Given these facts, the introduction of NPs into crop disease management programs represents a remarkable shift in modern agriculture systems. Their importance is underscored by the potential to enhance food security, promote sustainable agriculture, and reduce the environmental impact of conventional disease management methods. As research continues to uncover the intricacies of NPs in crop disease management, their roles in ensuring the health and resilience of global crop ecosystems become increasingly prominent. Different NPs and their potential in crop disease management are provided in Table 1.

**Table 1:** Selected examples of different NPs involved in the suppression of crop diseases through different mechanisms

Nanoparticles	Target pathogens	Host plants	Mechanisms	References
Copper	*Rhizoctonia solani*	Tomato	Suppressed disease progression by activating defense response	[31]
	*Acidovorax citrulli*	Watermelon	Activated stomatal immunity for disease suppression	[13]
	*Colletotrichum capsici*	Chilli	Reduced disease symptoms by directly inhibiting pathogen growth	[32]
Iron	*Tobacco mosaic virus*	Tobacco	Activated salicylic acid-dependent defense for suppressing viral infection	[33]
	*Ralstonia Solanacearum*	Tomato	Suppressed bacterial wilt by activating antioxidant enzymes and modulating rhizosphere bacterial community	[34]
Magnesium	*Colletotrichum gloeosporioides*	Avocado and papaya	Inhibited conidial germination and induced structural damage	[35]
	*R. solanacearum*	Tomato	Induced systemic resistance by triggering immune responses	[36]
Manganese	*Fusarium oxysporum* f. sp. *niveum*	Watermelon	Suppressed Fusarium wilt by inducing plant antioxidative and defense mechanisms	[4]
	*F. oxysporum* f. sp. *niveum*	Watermelon	Reduced disease incidence by triggering the expression of defense-related genes	[37]
Silver	*Fusarium oxysporum* f. sp. *lycopersici*	Tomato	Inhibited mycelial growth by inducing significant structural damages	[38]
	*Pectobacterium carotovorum*	Sugar beet	Activated antioxidative defense for suppressing soft rot disease	[39]
	*Xanthomonas oryzae* pv. *oryzae*	Rice	Inhibited disease incidence by activating plant antioxidative defense system	[40]
	*Acidovorax oryzae*	Rice	Inhibited pathogen survival, biofilm formation, and swarming motility	[41]
Sulfur	*F. oxysporum* f. sp. *lycopersici*	Tomato	Reduced disease incidence by activating salicylic acid-mediated disease resistance mechanisms	[27]
	*Fusarium solani*	Tomato	Disrupted pathogen cellular integrity and viability	[42]
	*P. carotovorum*	Lettuce	Decreased disease severity by triggering jasmonic acid- and salicylic acid-regulated immune responses	[43]
Titania	*Puccinia striiformis* f. sp. *tritici*	Wheat	Activated plant antioxidative signaling for suppressing fungal infection	[44]
	*Bipolaris sorokiniana*	Wheat	Reduced disease severity by improving plant physiological and metabolic profile	[45]
	*Dickeya dadantii*	Sweet potato	Inhibited pathogen growth, swimming motility, and biofilm formation	[46]
Zinc oxide	*X. oryzae* pv. *oryzae*	Rice	Showed direct antibacterial activity against bacterial pathogen	[28]
	*F. oxysporum* f. sp. *melongenae*	Eggplant	Suppressed disease severity by activating plant biochemical and physiological mechanisms	[47]
	*F. oxysporum* f. sp. *lycopersici*	Tomato	Reduced disease incidence by inducing defense responses	[48]

## Mechanisms of NPs-mediated crop disease management

**Fig. 1:**
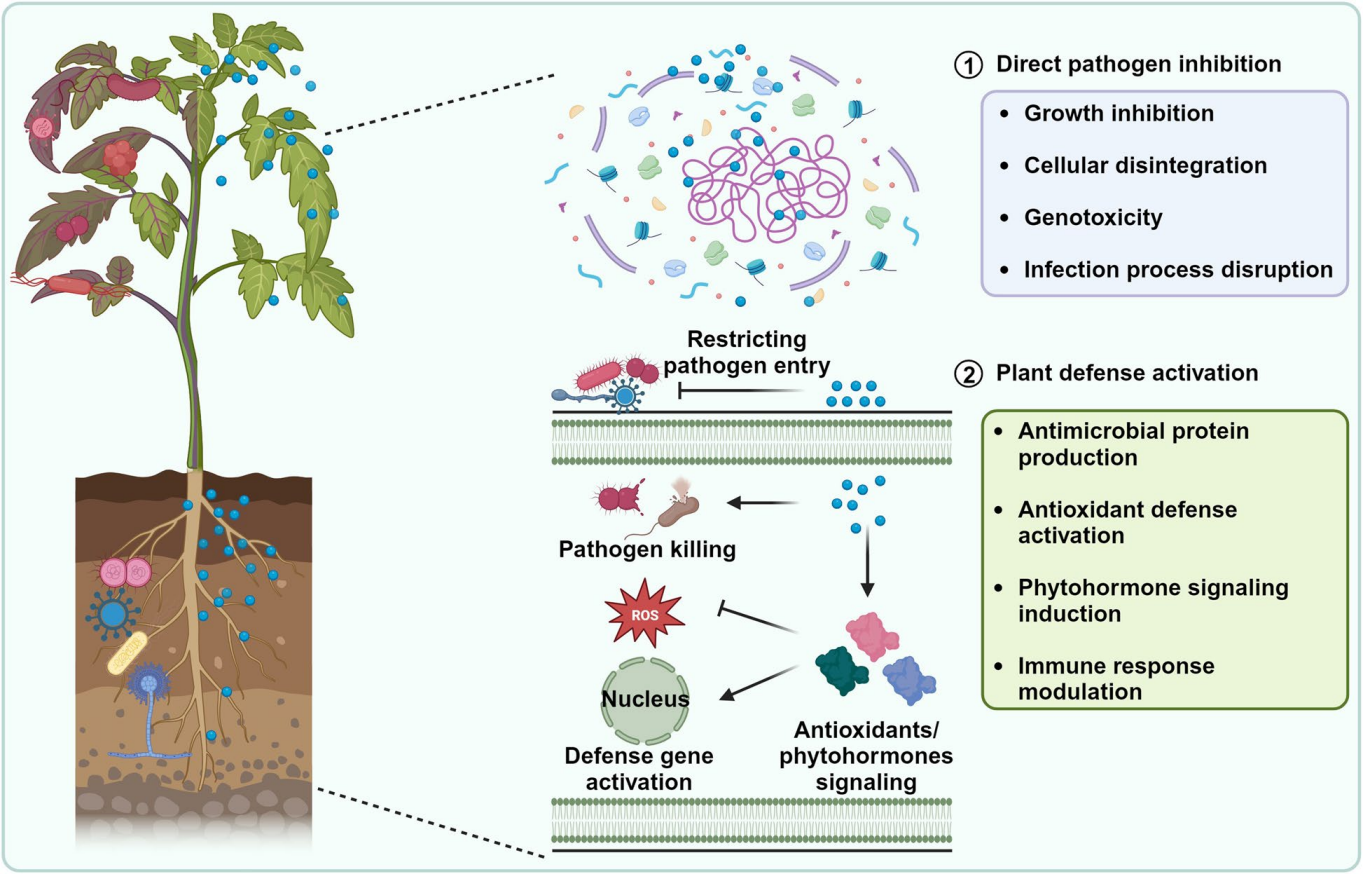
Mechanistic roles of NPs (*represented as blue-colored particles*) in crop disease management. NPs interact with phytopathogens either directly or indirectly. In direct interactions, NPs inhibit pathogen growth, reproduction, and infection processes, ultimately causing pathogen death. Through indirect mechanisms, NPs influx into plant cells and activate an intricate network of immune responses, including the production of antimicrobial metabolites, antioxidants, phytohormones, and pathogenesis-related proteins, thus providing resistance to plants against pathogen infection

NPs have emerged as versatile tools in the realm of disease management, offering novel approaches to combat phytopathogens and mitigate the devastating impacts of plant diseases (Fig. 1) [49]. The unique physicochemical properties of NPs, including small size, surface area-to-volume ratio, and catalytic potential, enable them to function as potent antimicrobial agents through disrupting pathogen growth, multiplication, and the infection process, and plant immunity modulators by regulating key defense-related pathways to improve resilience against pathogens [50]. Recent research progress has highlighted the multifaceted mechanisms of NPs in disease management and their effectiveness in countering phytopathogens (Table 1).

### NPs as antimicrobial agents

NPs possess inherent antimicrobial properties that can be harnessed to target a wide spectrum of pathogens. Their small size and large surface area facilitate interactions with microbial cells, leading to detrimental effects on their viability and function [51]. NPs, such as silver and copper NPs, are particularly noteworthy for their pronounced antimicrobial activity. These NPs can disrupt cell membranes, interfere with cellular processes, and induce oxidative stress, ultimately leading to the death of microbial cells [13, 40]. By functioning as antimicrobial agents, NPs can inhibit pathogen growth and disrupt the infection process, thus preventing disease onset and progression.

#### Disruption of pathogen structures and proliferation

NPs can exert inhibitory effects on the growth and proliferation of phytopathogens *via* disrupting cellular membranes and organelles, impeding their ability to establish infections and spread within plant tissues [14, 52]. The interactions between NPs and microbial pathogens can disrupt key physiological processes, including nutrient uptake, enzymatic activities, and cell division [53]. This disruption can lead to reduced pathogen populations and the mitigation of disease progression [10, 53]. For example, iron and copper nanocomposites, at 15 μg/mL and 32 μg/mL concentrations, respectively, have been shown to form a protective sheath on rice leaves, preventing *X. oryzae* pv. *oryzae* infection by disrupting its cellular structures and metabolic pathways [54, 55]. Such mechanisms offer the potential to limit the development of drug-resistant strains and enhance the durability of disease management strategies [56, 57]. Similarly, magnesium oxide NPs (16 μg/mL) have been found to interfere with cell division and inhibit spore germination in *Phytophthora nicotianae* and *Thielaviopsis basicola*, thereby curbing the proliferation of fungal pathogens. Furthermore, manganese and copper NPs (at 16 μg/mL and 100 μg/mL concentrations, respectively) have been reported to disrupt the integrity of essential biomolecules, such as nucleic acids and proteins, further attenuating pathogen growth [58, 59]. These NP-induced inhibitory effects hold immense promise for protecting plants against pathogen attack.

#### Inhibition of pathogen infection process

NPs can interfere with the intricate steps involved in the pathogen infection process, from adhesion to host tissues and chemotrophic/invasive growth to the establishment of infection structures [16]. By targeting specific stages of the infection process, NPs can impede the ability of pathogens to colonize and cause damage to host plants. NPs can hinder pathogen adhesion by modifying surface properties and repelling microbial cells [14, 60]. Furthermore, NPs can also disrupt the formation of biofilms, which is central to the establishment of many chronic infections [53, 61]. For example, chitosan-coated iron and magnesium nanocomposites (at 250 μg/mL and 100 μg/mL concentrations, respectively) have shown promise in preventing biofilm formation by devastating rice bacterial pathogens, *X. oryzae* pv. *oryzae* and *Acidovorax oryzae*, where chitosan facilitated greater influx of nano-iron and magnesium into microbial cells, thus enhancing their antimicrobial potential and attenuating infection on plant surfaces [14, 62]. Manganese, copper, and sulfur NPs have been shown to alter the physicochemical properties of plant surfaces, making them less conducive to pathogen attachment and colonization [4, 13, 27]. Although this disruption of the infection process holds potential for mitigating disease incidence and severity, challenges such as optimizing NP efficacy, understanding potential ecological impacts, and ensuring targeted delivery remain to be addressed. As research in this field continues, the application of NPs could revolutionize disease management practices, contributing to the development of resilient and sustainable agricultural systems.

### NPs as modulators of plant immunity

Plants have evolved an intricate innate immune system to defend against phytopathogenic invaders, underscoring the significance of plant autoimmunity in safeguarding their health [63, 64]. The use of NPs as immune modulators offers a novel approach to bolster plant immune responses, potentially leading to enhanced broad-spectrum resistance against a wide range of pathogens. NPs can effectively trigger and amplify various aspects of plant defenses, encompassing both early and late immune signaling and defense responses [65]. Recent studies have highlighted different mechanisms by which NPs modulate plant immune responses to suppress diseases in crops (Table 1).

#### Reinforcement of physical barriers

The application of NPs to enhance physical barriers in crops represents a cutting-edge strategy in improving plant defenses against pathogens. Physical barriers serve as the first line of defense, preventing pathogen invasion by inhibiting attachment, penetration, and colonization. NPs have emerged as promising agents to reinforce these barriers, providing an additional layer of protection.

One of the primary mechanisms through which NPs reinforce physical barriers is by modifying plant surfaces to make them less suitable for pathogen attachment [66]. For example, manganese NPs (100 μg/mL concentration) have demonstrated the ability to alter watermelon root surfaces to restrict *F. oxysporum* f. sp. *niveum* entry. These NPs formed a nanotextured layer on the root surface, effectively reducing pathogen penetration and infection in watermelon plants [4]. Furthermore, NPs can enhance the structural integrity of plant cell walls and cuticles, fortifying physical barriers against pathogen penetration [67]. Cell walls act as a formidable physical barrier, and silica NPs have been reported to act as a cell wall reinforcing agent, reducing *Pseudomonas syringae* pv. tomato (*Pst*) DC3000 growth in *Arabidopsis thaliana* [68]. Furthermore, this reinforcement impedes the progress of invading pathogens, reducing infection severity [69]. NPs also play a role in the modification of plant trichomes, specialized epidermal structures that can serve as physical barriers against devastating pathogens [16, 70]. Silica and copper NPs, at 25 mg/L and 100 μg/mL concentrations, respectively, have been shown to maintain the integrity of trichomes in soybean and watermelon plants, providing enhanced protection against *Fusarium virguliforme* and *Acidovorax citrulli* infections, respectively [13, 16].

The importance of NPs in reinforcing physical barriers lies in their ability to enhance crop resilience. By minimizing pathogen attachment, penetration, and subsequent colonization, NPs help reduce disease incidence and severity. This not only leads to increased crop yield but also lowers the need for chemical pesticides, aligning with sustainable and environmentally friendly agricultural practices [49, 71, 72]. However, challenges remain, particularly in fine-tuning the application of NPs to achieve optimal barrier reinforcement without negative side effects on plant growth and development. Additionally, understanding the long-term implications of NPs on the overall health of crops and ecosystems is a vital area of future research.

#### Elicitation of phytohormone signaling

The manipulation of phytohormone signaling pathways through the use of NPs has emerged as a pioneering strategy to fortify crops against pathogenic threats [73]. Phytohormones, such as salicylic acid (SA) and jasmonic acid (JA), play pivotal roles in orchestrating plant defense mechanisms [74–76]. NPs have been demonstrated to activate plant immune responses by modulating the levels and signaling pathways of phytohormones [7, 73, 77, 78].

Although the intricate network of NPs and phytohormone signaling is unexplored, studies have demonstrated that NPs and hormonal signaling synergistically regulate defense mechanisms in plants under pathogen attack [79, 80]. For example, copper NPs at 50 mg/L have been shown to enhance SA- and JA-mediated defenses, resulting in increased resistance in rice against the bakanae disease-causing fungal pathogen *Gibberella fujikuroi* [81]. Similarly, silver NPs at 50 μg/mL have been reported to induce SA-mediated defense responses in wheat plants, providing protection against the yellow rust pathogen *Puccinia striiformis* f. sp. *tritici* [82].

This innovative approach of NPs-mediated elicitation of phytohormone signaling pathways offers several advantages in crop protection. First, it enables plants to fine-tune their responses according to the type of pathogen encountered. Second, it facilitates rapid and robust defense responses, reducing the severity of disease symptoms. Third, it allows for a fine-tuned balance between growth and defense, ensuring minimal impact on plant development [73, 83, 84]. However, the use of NPs to modulate phytohormone signaling is not without its challenges. Achieving precise control over the desired hormonal responses and minimizing potential side effects are ongoing areas of research [73]. It is believed that as the understanding of NP-phytohormone interaction networks deepens, their potential to bolster crop resilience and secure global food production becomes increasingly evident.

#### Production of antimicrobial compounds

Harnessing the potential of NPs to facilitate the production of antimicrobial compounds, such as pathogen-fighting molecules and enzymes, within crops represents a pioneering approach to fortify plant defenses against pathogens [85]. Antimicrobial compounds are key components of a plant arsenal against invading microbes, as they can directly inhibit pathogen growth and multiplication [86, 87]. NPs offer a remarkable means to stimulate the biosynthesis of these compounds, equipping plants with enhanced resistance.

NPs can stimulate the synthesis of antimicrobial compounds, such as pathogenesis-related (PR) proteins, phenolic compounds, and hydrolytic enzymes, contributing to plant defense [85, 88]. These compounds exhibit inhibitory effects on pathogens, impeding their growth and infection process [89, 90]. Sulfur NPs (30 mg/L) have been found to induce the expression of PR and antioxidant enzyme-encoding genes in tomato, resulting in increased resistance to Fusarium wilt pathogen *Fusarium oxysporum* f. sp. *lycopersici* [27]. Additionally, silica NPs at 650 mg/L have been shown to elevate the production of antimicrobial hydrolytic enzymes, such as *β*-1,3-glucanase, pectinase, and chitinase, in tomato plants against the bacterial wilt pathogen *Ralstonia solanacearum* [91].

The significance of NPs in promoting the production of antimicrobial compounds lies in their potential to provide crops with a proactive and self-sustaining defense system. By stimulating the biosynthesis of antimicrobial compounds, NPs empower plants to produce their own arsenal of weapons to combat pathogens. This not only reduces disease incidence but also decreases the need for external chemical interventions, contributing to eco-friendly and sustainable agriculture [85, 88]. However, the use of NPs to induce antimicrobial compound production in crops is a promising approach, with considerations related to optimal dosage, application timing, and potential side effects on plant growth and development. Moreover, understanding the long-term effects of NPs on crop health and ecosystem dynamics is essential for responsible applications.

#### Activation of systemic acquired resistance

Harnessing NPs to activate systemic acquired resistance (SAR) in crops marks a revolutionary approach to bolster plant immunity against a spectrum of pathogens. SAR is a sophisticated defense mechanism in plants that primes their entire structure for enhanced resistance to subsequent pathogen attacks [92, 93]. NPs have emerged as promising agents to induce SAR, providing crops with a durable and comprehensive shield against pathogens.

The fundamental mechanism underlying NPs-mediated SAR induction is the modulation of signaling pathways within the plant [94]. When NPs interact with crops, they can trigger the production of signaling molecules, such as SA, which play pivotal roles in orchestrating plant defense responses [95]. For instance, 10 mg/L nitrogen-doped carbon NPs have been reported to trigger SA- and JA-dependent SAR responses in tomato plants, suppressing bacterial wilt disease by reducing *in planta* pathogen growth [96]. Moreover, silver-silica nanocomposites at 10 μg/mL triggered the expression of the defense genes *PR1*, *PR2*, and *PR5* in *Pst* DC3000-challenged *Arabidopsis* plants [97], suggesting that NPs can induce SAR in plants *via* regulating defense signaling pathways.

By priming plants for SAR, NPs equip them with an enduring defense mechanism, allowing for rapid and effective responses to subsequent pathogen encounters [98]. This not only reduces disease incidence but also lowers the need for chemical pesticides, promoting sustainable and environmentally friendly agricultural practices. However, it is essential to consider the complexities of NP-plant interactions, including dose-dependent effects and potential crosstalk between defense pathways, to optimize the elicitation of plant defense responses. As NP-mediated plant immune modulation continues to be explored, a deeper understanding of the underlying mechanisms will undoubtedly contribute to the advancement of innovative approaches in plant disease management.

## Mechanisms of NP uptake by plants

**Fig. 2:**
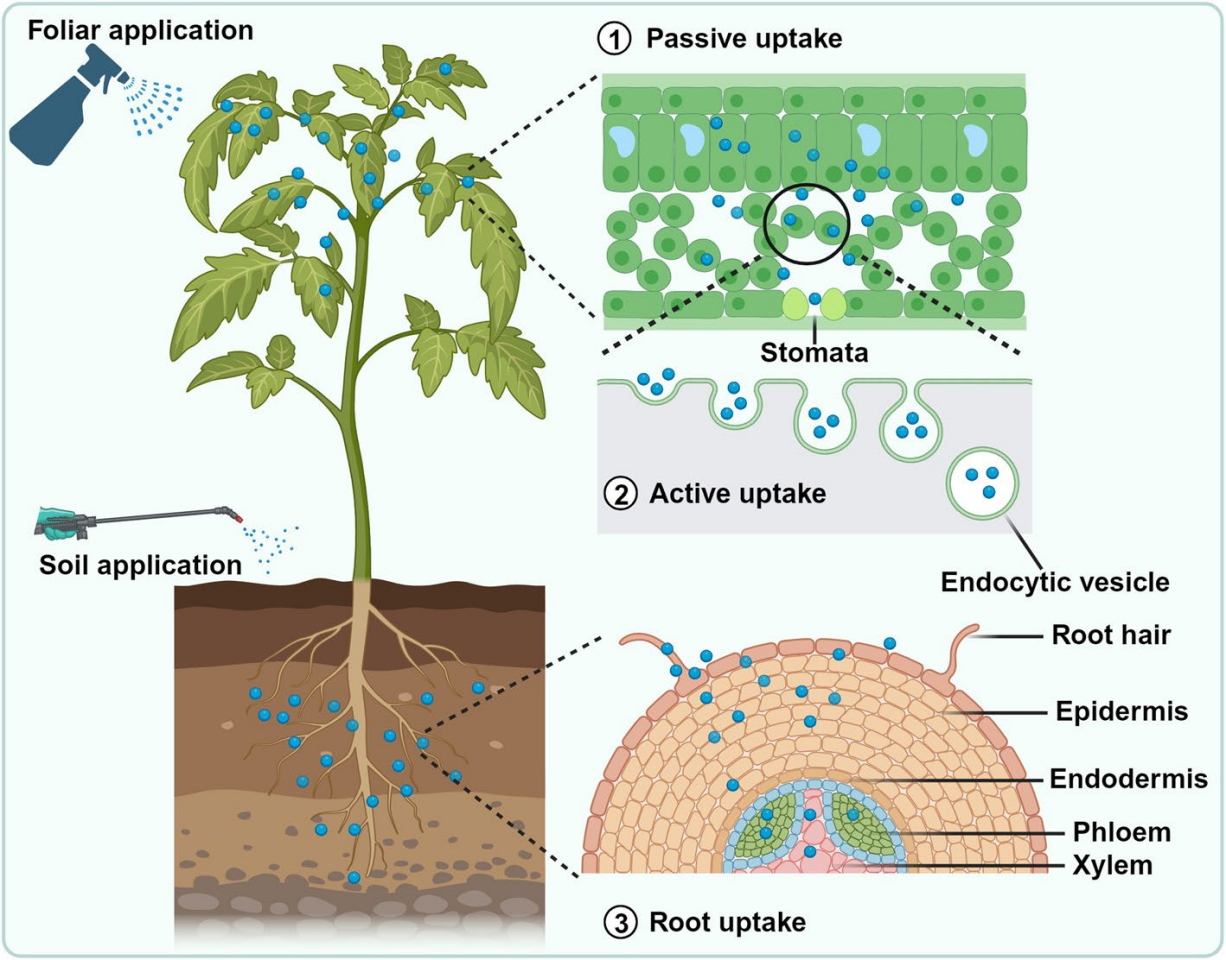
Modes for the application and uptake of NPs (*represented as blue-colored particles*) in plants. When applied through foliar application, plants uptake NPs passively through stomatal openings and then internalize them into cells actively through endocytosis. Plants uptake NPs through their roots following soil-based application and then translocate NPs into xylem and phloem tissues from where they are transported to aerial parts

Understanding NP uptake by plants provides crucial insights into how NPs interact with plant cells and activate immune responses [99]. Several pathways have been proposed to elucidate how NPs are internalized by plants (Fig. 2), facilitating their interaction with key immune signaling components [6, 43, 92]. Accumulating evidence has revealed different NP uptake mechanisms that are commonly used by plants.

### Passive uptake of NPs

NPs can be passively taken up by plants through processes such as diffusion and adsorption [100]. These mechanisms primarily depend on the physicochemical properties of NPs, including size, surface charge, and hydrophobicity [101]. Once NPs come into contact with the plant surface, they may diffuse through the cuticle or stomata and accumulate in various plant tissues [102, 103]. The hydrophobic nature of many metallic NP surfaces enables them to traverse the lipophilic cuticle, especially when in contact with water. Additionally, NPs can enter the plant through stomatal openings, which serve as entry points for various materials, including NPs [104, 105]. For example, polyvinylpyrrolidone- and citrate-coated gold NPs are passively taken up by wheat leaves *via the* cuticular pathway [103]. The small size and unique surface properties of gold NPs enable their diffusion through plant surfaces, leading to their internalization. However, further research is required to understand the downstream translocation mechanisms of foliar NPs through phloem.

### Active uptake of NPs

Active uptake mechanisms enable plants to selectively internalize NPs, ensuring precise targeting and controlled responses [106]. NPs can be taken up actively by plant cells through endocytosis, a process in which the cell membrane engulfs the NPs to form vesicles [107]. This process is common in plants and allows them to internalize NPs enclosed within vesicles. Endocytosis provides a means for plants to regulate the uptake of NPs and respond to environmental cues [108]. Although the mechanism of NP internalization by plants is not well understood, previous studies have shown that clathrin-mediated or caveolin-mediated endocytosis pathways actively facilitate the uptake of NPs into plant cells. For example, gold NPs are actively taken up by tobacco protoplasts through clathrin-dependent pathways [109]. The superficial properties of NPs play crucial roles in their uptake by plant cells through endocytosis. For example, tobacco mesophyll protoplasts internalized triethylene glycol-functionalized silica NPs, while bare un-functionalized silica NPs did not enter the plant cells [110]. Despite these facts, direct evidence regarding endocytic NP uptake by plant cells is still lacking. Thus, future studies should be focused on investigating integrated mechanisms of NP uptake and translocation, which will enlighten our understanding regarding how NP uptake and translocation occur within the plant system.

### Root uptake and translocation of NPs

Soil-applied NPs primarily enter plants through the roots, where they encounter root hairs and epidermal cells [111, 112]. NPs that are positively charged or exhibit specific chemical interactions with root cell walls tend to adhere to and enter plant roots more readily [113, 114]. Once inside the root, NPs can be translocated to other plant parts through the xylem and phloem [6, 115]. It has been shown that the transpiration rate positively influences NP uptake by plant roots. For example, transmission electron microscopy and energy dispersive analysis of xylem revealed that copper NPs travelled along with the water in the xylem, facilitating their translocation from roots to aerial parts of maize [116].

The versatility of NP uptake pathways offers avenues for targeted delivery and controlled release of bioactive compounds, thereby enhancing their potential as effective plant immune modulators [117]. However, various factors, including the physicochemical properties of NPs, their intended purpose, targeted crops, and the desired mode of interaction with the plant system, are crucial in governing the selection of their application methods and performance in the agriculture sector [6]. As research in this area continues to advance, a deeper understanding of NP uptake mechanisms holds the key to unlocking their full potential in sustainable plant disease management.

## NPs as delivery vehicles for bioactive molecules

NPs, with their unique physicochemical properties and tunable surfaces, have emerged as promising carriers for a variety of bioactive compounds [118], e.g., essential nutrients, plant hormones, RNA interference (RNAi) molecules, and chemical protectants. This feature of NPs sheds light on their potential to revolutionize nutrient management, growth regulation, and genetic manipulation in plants.

### Delivery of essential nutrients

NPs have garnered significant attention as carriers for delivering essential nutrients to plants, ensuring their optimal uptake and utilization. By encapsulating nutrients within NPs, their solubility, stability, and bioavailability can be enhanced, overcoming challenges associated with conventional nutrient delivery methods [119, 120]. For example, iron, manganese, copper, and zinc deficiencies are prevalent in many crops, leading to reduced yield and nutritional quality [121]. Nanofertilizers have been designed to enhance nutrient absorption by plants [122]. These NPs protect nutrients from degradation and release them in a controlled manner, facilitating efficient uptake by plant tissues [123, 124]. The use of NPs as nutrient carriers holds promise for alleviating nutrient deficiencies and enhancing crop productivity, thus addressing global food security challenges [125]. For example, zinc-based nanofertilizers have been shown to promote seed germination and growth in a variety of crops, such as wheat, onions, peanuts, and soybean [126]. Furthermore, copper oxide NPs have been reported to efficiently deliver copper to tomato and eggplant seedlings, suppressing Fusarium wilt while improving nutritional quality and crop yields [127]. Similarly, copper NPs suppressed Fusarium wilt and bacterial fruit blotch in watermelon plants *via* improving copper accumulation in plant tissues [13, 128]. Although nanofertilizers have shown promising roles in improving plant growth and health under greenhouse conditions, field-scale trials need to be conducted to assess their plant growth-promoting potential under real environmental conditions.

### Delivery of plant hormones

Plant hormones play a pivotal role in regulating various physiological processes, including growth, development, and defense responses [129]. NPs offer a sophisticated platform for the targeted delivery of plant hormones, enabling precise manipulation of plant behavior against infection or disease [130, 131]. For instance, abscisic acid (ABA) is a hormone involved in stress responses and water regulation. Mesoporous silica NPs have been designed for the delivery of ABA to *Arabidopsis* [132]. In a recent study, biogenic iron NPs were employed for therapeutic delivery of SA into watermelon plants, providing enhanced resistance against Fusarium wilt disease *via* activating SA-mediated immune responses [7]. The nano-enabled smart delivery system ensures that plants receive hormone signals exactly when needed, optimizing their stress tolerance and resource utilization [133]. Similarly, chitosan-based nanocomposites can be engineered to deliver growth-promoting hormones, including auxins and gibberellic acid, promoting root development, shoot growth, and flowering in agronomically important plants [134]. Harnessing NPs for hormone delivery represents opportunities to fine-tune plant growth and stress responses, contributing to enhanced crop performance and resilience.

### Delivery of small-interfering RNAs

The advent of RNAi technology has revolutionized genetic manipulation and crop improvement [135]. NPs offer a versatile platform for delivering small-interfering RNAs (siRNAs), which mediate RNAi responses, directly into plant cells, providing resistance against pests or pathogens [136]. This capability enables targeted gene silencing, allowing researchers to modulate the expression of specific genes associated with disease susceptibility, insect resistance, or other agronomically important traits [137]. NPs protect siRNAs from degradation in the harsh extracellular environment and facilitate their cellular uptake, enhancing the efficiency of gene silencing [22]. Previously, pathogen-specific double-stranded (ds)RNA delivered through layered double hydroxide nanosheets showed stability for up to 30 days and provided long-term protection to tobacco plants against viral infections compared with naked dsRNA [138]. Thus, this approach has shown promise in conferring resistance against pathogens and pests, reducing the need for chemical interventions. The use of NPs as siRNA carriers provides a powerful tool for precision agriculture and the development of sustainable crop protection strategies [139].

### Delivery of chemical protectants

The utilization of NPs as delivery vehicles for chemical protectants in crops heralds a revolution in precision agriculture and disease management [140]. Chemical protectants, such as fungicides, bactericides, and other antimicrobial agents, are vital tools in safeguarding crops from the devastating impact of phytopathogens [141]. However, their efficient and targeted delivery has long been a challenge. In this regard, NPs have emerged as a game-changing solution. By encapsulating or binding these chemical protectants, NPs offer a precise and controlled means of delivering them to specific plant tissues and pathogens [140]. Previously, mesoporous silica NPs have been employed as a delivery carrier for prochloraz, providing longer and better protection against rice blast disease [142]. Similarly, azoxystrobin-loaded silica NPs exhibited better fungicidal activity against the tomoto late blight pathogen *Phytophthora infestans* than the regular form of fungicide [143]. Nanoscale fungicides, fenhexamid and polyhexamethylene biguanide, showed better antimicrobial activity against devastating crop pathogens, *Pseudomonas syringae* pv. *lachrymans*, *Botrytis cinerea*, and *Sclerotinia sclerotiorum* than their bulk forms [144].

Furthermore, NPs can be engineered to release their cargo in response to specific triggers or environmental conditions [145]. For instance, pH-sensitive carbendazim-loaded mesoporous selenium NPs have been synthesized against *S. sclerotiorum*, ensuring target-specific fungicide delivery under acidic conditions [146]. This targeted delivery minimizes off-target effects and reduces the overall amount of fungicides needed, promoting eco-friendly and sustainable agricultural practices. Although the delivery of chemical protectants *via* NPs represents a transformative approach to disease management in agriculture, several challenges and considerations, including safety concerns and non-target effects on organisms or the environment, need to be assessed.

Overall, NPs have emerged as versatile delivery vehicles for bioactive molecules in plants, offering a novel means of addressing agricultural challenges. By encapsulating essential nutrients, plant hormones, siRNAs, and chemical protectants, NPs enable targeted and controlled release, enhancing nutrient availability, growth regulation, and genetic manipulation. As research in nanotechnology advances, the potential applications of NP-mediated smart delivery systems continue to expand, offering innovative solutions to improve crop productivity, nutritional value, and sustainability. However, challenges such as NP biocompatibility, environmental impact, and regulatory considerations need to be carefully addressed as this technology evolves toward practical implementation in agriculture.

## Safety and environmental considerations of NP application

NPs have gained significant interest for their potential applications in various fields, including agriculture. While their unique properties offer exciting possibilities, the safety and environmental implications of NP use must be thoroughly examined to ensure responsible and sustainable deployment [147]. In this context, the safety issues described below, such as ecotoxicity, environmental fate, and regulatory aspects, associated with NP use in agriculture must be taken into consideration.

### Toxicity and ecotoxicity

The potential toxicity of NPs is a central concern that warrants rigorous evaluation. NPs can interact with biological systems, and their small size and high surface area-to-volume ratio may lead to unique interactions with living organisms [148]. Understanding the potential adverse effects of NPs on plants, non-target organisms, and ecosystems is paramount. Studies have shown that NPs, when present in excessive concentrations, can disrupt cellular processes, impair plant growth, and affect soil microbial communities [149–151]. For example, zinc oxide and cerium oxide NPs have been shown to induce genotoxicity in soybean, inhibiting plant growth and development [152]. Furthermore, iron oxide and carbon NPs have been reported to negatively impact soil bacterial abundance and shift community composition by reducing soil dissolved organic carbon contents [149, 150]. Efforts to assess nanotoxicity involve examining cellular responses, physiological changes, and long-term effects on plant health. Furthermore, investigating the bioaccumulation and biomagnification potential of NPs within food chains is essential to predict their impact on higher trophic levels [153].

### Environmental fate and transport

NPs released into the environment may undergo transformations that influence their behavior, mobility, and potential impact [154]. Factors such as particle size, surface chemistry, and environmental conditions can affect NP fate and transport [155, 156]. Understanding the mechanisms governing NP interactions with soil, water, and air is crucial for predicting their distribution and potential migration to water bodies or uptake by plants. NPs may also adsorb onto soil particles, altering soil properties and influencing nutrient availability [157]. Although NPs-based disease management approaches have shown tremendous potential, it is crucial to devise a pathway for their safe disposal after use. Recently, it has been shown that certain NPs can undergo biodegradation or transformation into simpler less-reactive forms [158]. For example, NADPH oxidase-mediated biodegradation of gold NPs has recently been reported [159]. Given that next-generation nanopesticides with inherent biodegradation ability or advanced filtration techniques need to be designed to enhance the removal of residual NPs from the environment. Furthermore, comprehensive studies on the environmental fate of NPs are pivotal to assess their long-term persistence, potential for dispersion, and likelihood of unintended accumulation.

### Risk assessment and mitigation

Robust risk assessment frameworks are essential to guide the safe implementation of NP-based technologies in agriculture [160]. Integrated approaches, combining laboratory studies, field trials, and modeling, are necessary to comprehensively evaluate potential risks. The development of standardized protocols for NP characterization, toxicity testing, and environmental monitoring is crucial for generating reliable data for risk assessments [161]. In cases where NPs exhibit adverse effects, mitigation strategies can be explored, such as modifying NP properties to reduce toxicity or enhancing NP retention within the target plant tissues [162, 163].

### Regulatory considerations

The introduction of NP-based products into agriculture necessitates a robust regulatory framework that ensures both innovation and safety [164, 165]. Regulatory agencies must work collaboratively with researchers, industries, and stakeholders to establish guidelines for NP use in agriculture. Transparent reporting of NP properties, toxicity data, and environmental impact assessments is crucial for informed decision-making [148, 157]. International cooperation and harmonization of regulations will facilitate the responsible integration of NPs into agricultural practices while minimizing potential risks.

## Future perspectives and concluding remarks

NPs have emerged as a transformative force in reshaping the landscape of plant science and agriculture. As our understanding of their interactions with plants and phytopathogens deepens and as technological advancements continue to accelerate, the future holds immense promise for the integration of NPs into sustainable agricultural practices. The future perspectives and overarching themes for the application of NPs in crop disease management will be focused on the following aspects.

### Precision agriculture revolution

The integration of NPs into precision agriculture holds the potential to revolutionize crop management [120]. By precisely targeting specific plant responses, such as immune modulation, nutrient delivery, and growth regulation, NPs enable a level of precision that was previously unthinkable [166, 167]. This precision-driven approach could lead to optimized resource utilization, reduced environmental impact, and improved crop yields [106]. Furthermore, the development of “smart” nanoagrochemicals that respond to environmental cues could usher in an era of dynamic and adaptable crop management strategies tailored to the unique needs of each plant and field.

### Eco-friendly disease management

NPs have the potential to significantly reduce the reliance on conventional chemical pesticides, offering a more sustainable approach to disease management [168]. As NPs demonstrate efficacy in inhibiting pathogen growth, disrupting infection processes, and enhancing plant defense responses, they can be harnessed to develop eco-friendly alternatives [169]. The reduction in chemical pesticide use not only benefits the environment but also addresses concerns related to pesticide resistance and food safety [6]. The future may witness NP-based formulations replacing or complementing traditional crop disease management practices, leading to more resilient and healthier crop ecosystems.

### Unraveling plant-NP interactions

Advancements in nanotechnology are poised to provide deeper insights into the intricate interactions between NPs and plants. As our understanding of NP uptake, transport, and mechanisms of action expands [5, 112, 170–172], we can anticipate the discovery of new pathways for enhancing plant health and resilience. Cutting-edge techniques such as high-resolution imaging, omics analyses, and computational modeling will enable us to decipher the molecular and physiological changes induced by NPs. This knowledge will guide the design of NPs with tailored properties optimized for specific plant‒microbe interactions, unlocking their full potential for sustainable disease management and crop improvement.

### Balancing innovation with responsibility

Although the potential of NPs is vast, responsible innovation is paramount. Addressing safety, environmental, and regulatory concerns will be essential to ensure that NP applications do not inadvertently introduce unintended consequences [6, 173]. A multidisciplinary approach involving researchers, regulators, policymakers, and stakeholders will be crucial to strike a balance between technological advancement and the preservation of ecosystems. Open dialogue and collaborative efforts will guide the development of guidelines and frameworks for the safe and sustainable use of NPs in agriculture.

In conclusion, the integration of NPs into the crop disease management system represents a paradigm shift with transformative implications for agriculture. By harnessing the unique properties of NPs, we stand poised to enhance plant immunity, nutrient uptake, and growth regulation in an environmentally friendly manner. As we navigate the exciting prospects that lie ahead, it is imperative to approach NP research and application with a holistic and responsible perspective. By doing so, we can pave the way for a future where NPs contribute to sustainable, resilient, and productive agricultural systems, fostering a brighter and more secure future for global food production.

## Data Availability

All data used in this study are included in this article.
